# One-Step Biosynthesis of Soft Magnetic Bacterial Cellulose Spheres with Localized Nanoparticle Functionalization

**DOI:** 10.1021/acsami.1c17752

**Published:** 2021-11-12

**Authors:** Soledad Roig-Sanchez, Oriol Torrecilla, Jordi Floriach-Clark, Sebastià Parets, Pavel A. Levkin, Anna Roig, Anna Laromaine

**Affiliations:** † Institut de Ciència de Materials de Barcelona, ICMAB-CSIC, Campus UAB, Bellaterra, Barcelona 08193, Spain; ‡ Institute of Biological and Chemical Systems-Functional Molecular Systems (IBCS-FMS), Karlsruhe Institute of Technology (KIT), Eggenstein-Leopoldshafen 76344, Germany

**Keywords:** 3D structure, magnetic bacterial cellulose, actuator, nanocomposite, Janus sphere, SPIONs, nanoparticles

## Abstract

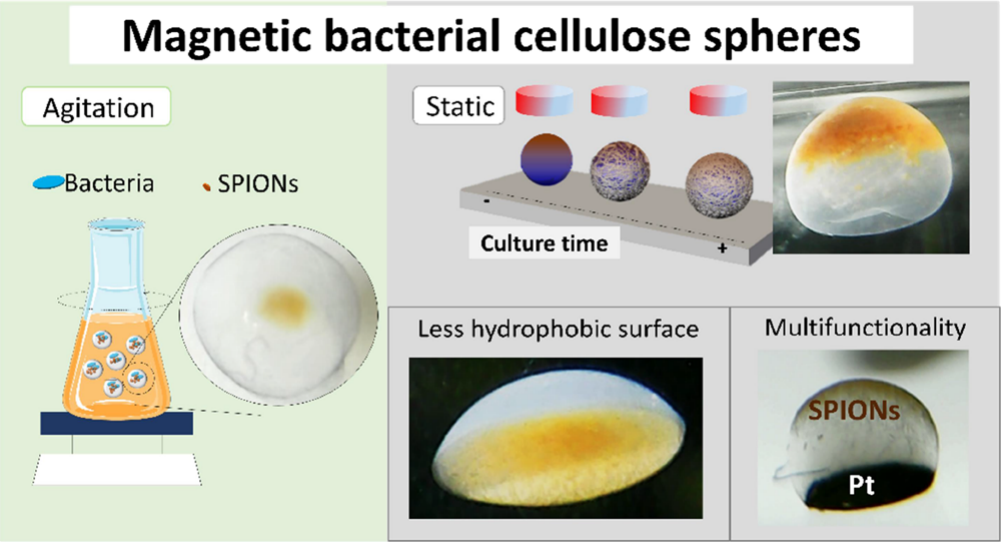

Actuated structures are becoming relevant in medical fields; however, they call for flexible/soft-base materials that comply with biological tissues and can be synthesized in simple fabrication steps. In this work, we extend the palette of techniques to afford soft, actuable spherical structures taking advantage of the biosynthesis process of bacterial cellulose. Bacterial cellulose spheres (BCS) with localized magnetic nanoparticles (NPs) have been biosynthesized using two different one-pot processes: in agitation and on hydrophobic surface-supported static culture, achieving core-shell or hollow spheres, respectively. Magnetic actuability is conferred by superparamagnetic iron oxide NPs (SPIONs), and their location within the structure was finely tuned with high precision. The size, structure, flexibility and magnetic response of the spheres have been characterized. In addition, the versatility of the methodology allows us to produce actuated spherical structures adding other NPs (Au and Pt) in specific locations, creating Janus structures. The combination of Pt NPs and SPIONs provides moving composite structures driven both by a magnetic field and a H_2_O_2_ oxidation reaction. Janus Pt/SPIONs increased by five times the directionality and movement of these structures in comparison to the controls.

## Introduction

Bacterial cellulose (BC) is a biopolymeric hydrogel made of intertwined nanocellulose fibrils secreted by bacteria, such as *Komagateibacter xylinus*, at the air interface of the liquid culture. This low-density hydrogel that exhibits a porous fibrillary network, which confers a high water-holding capacity, is highly transparent and insoluble in water. BC also reveals remarkable robust mechanical properties, biodegradability, and biocompatibility.^1−3^ Altogether, these attributes raise BC composites as valuable candidates to assemble them as soft actuators.

Soft actuators are attractive tools in medicine; however, they have to comply with biomedical requirements, such as biocompatibility, flexibility, self-healing, and adaptation to different biological environments.^4−6^ Actuators are usually built from a base material, and actuation or movement is conferred by a modification of the structure or by the addition of an auxiliary material to create stimulus-responsive composites.^7−10^ Among the current palette of actuators’ base materials, polymers arise as strong candidates. They are flexible and resilient to fracture, lightweight, their shape can be adapted during manufacture and they can be efficiently loaded with drugs;^11−14^ a set of characteristics rarely achieved using traditional rigid actuators.^15^

Actuators commonly reported mimic bacteria or present a spherical structure. Within spheres, silica^16^ or poly(lactic-*co*-glycolic acid) (PLGA)^17^—based actuators are extensively used as cargo-delivery systems, theragnostic, tissue engineering or metal-ion sensing.^16−20^ Despite the clear suitability of BC spheres (BCS), they have only been proposed for bioseparation, heavy-metal ion removal and immobilization reactions due to their large surface areas.^21^ Additionally, BC can be functionalized by the incorporation of additives ranging from conductive polymers (as polyaniline^22^ or PEDOT^23^), biopolymers,^24^ carbon-based materials (as graphene^25^ or carbon nanotubes^26^), and ceramics (hydroxyapatite^27^ or silica^28^) to metallic nanoparticles (NPs),^29,30^ displaying ancillary properties or extra response to different stimuli, such as magnetic, optical, catalytic, anti-inflammatory, antioxidant, or antimicrobial properties among others.^31^

Here, we evaluate magnetic BCS (core-shell and hollow) as actuators produced in a single biosynthesis step through agitation or by static bacterial culture over hydrophobic surfaces. The placement of superparamagnetic iron oxide NPs (SPIONs) within the spherical structure is successfully controlled by local magnetic fields. Several strategies have been reported to produce BC spheres such as three-dimensional (3D) printing,^32^ microfluidics giving rise to hollow BC microspheres^33^ or spray-drying, yielding BC microparticles;^34^ however, specialized equipment is required in all these cases and localized functionalization, and, in particular, multifunctionalization of these spheres is scarce.^25,35^ Our novel static culture method allowed the production offlexible magnetic BCS controlling the NP loading position within the sphere, a result not reached previously. In addition, we have produced size-controlled Janus BC structures in the same biosynthesis single step, the propelling ability of which has been tested under a different stimuli, such as magnetic fields and media acting as fuel.

## Results and Discussion

**Figure 1:**
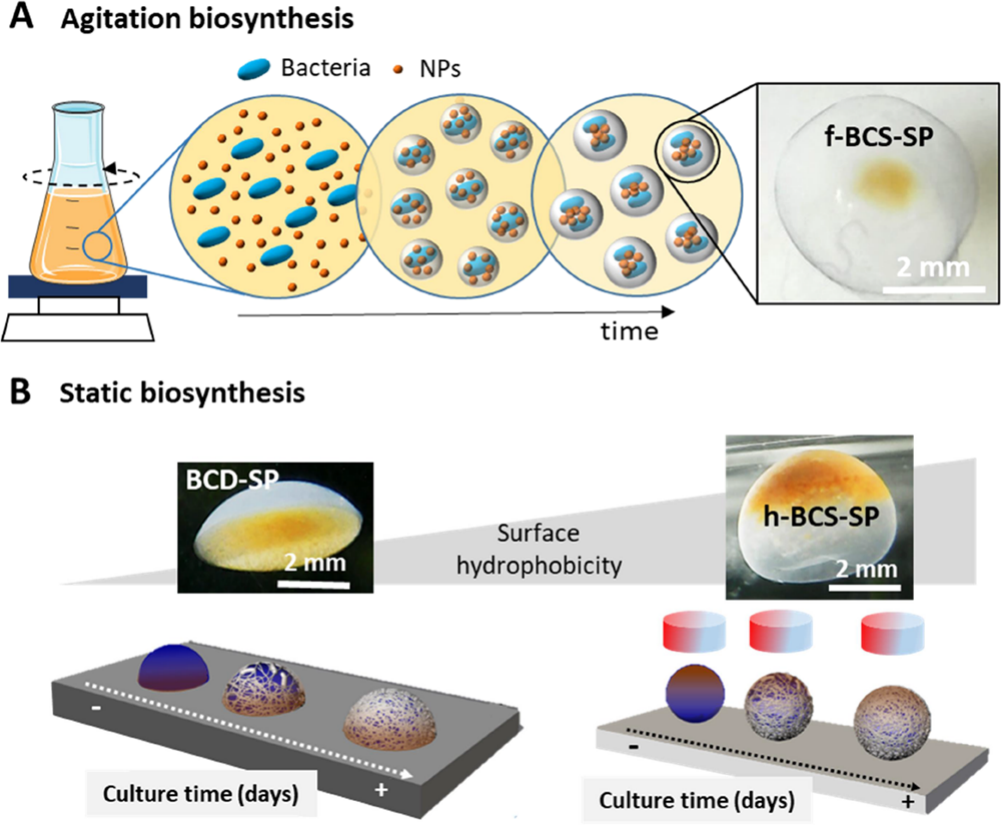
(A) Scheme of the agitation biosynthesis method to produce filled bacterial cellulose spheres with magnetic NPs in the center (f-BCS-SP). Picture of an f-BCS-SP where the core-shell structure is appreciated. (B) Scheme of the static biosynthesis process for hollow bacterial cellulose spheres (h-BCS) production where the shape is controlled by the surface hydrophobicity and the placement of SPIONs by application of an oriented magnetic field. Left: Picture of a hollow dome structure with magnetic NPs at the bottom (BCD-SP). Right: Picture of a hollow sphere with magnetic NPs (SPIONs) on top (h-BCS-SP).

We have produced functional BCS exploiting two accessible biosynthesis approaches: in agitation and in static conditions over hydrophobic surfaces. In the first case, the formation of BC occurs in the bacterial culture at 30 °C under constant agitation. As described by Hu and coworkers,^36^ bacteria aggregate due to stirring and produce nanofibers, creating skein-like structures trapping the bacteria and achieving filled BCS (f-BCS). Their size and shape are influenced by additional factors in addition to the bacteria concentration, temperature, carbon, or nitrogen source such as the stirring speed or the oxygen content.^21,37^ To provide functionality to f-BCS, SPIONs were added to the culture medium to attain magnetic spheres (Figure 1A). For this purpose, monodisperse SPIONs of 7.1 ± 1.5 nm in diameter (polydispersity index, PDI: 21%) were synthesized in-house using a previously described procedure,^29^ and the characterization is provided in Figure S1.

**Figure 2:**
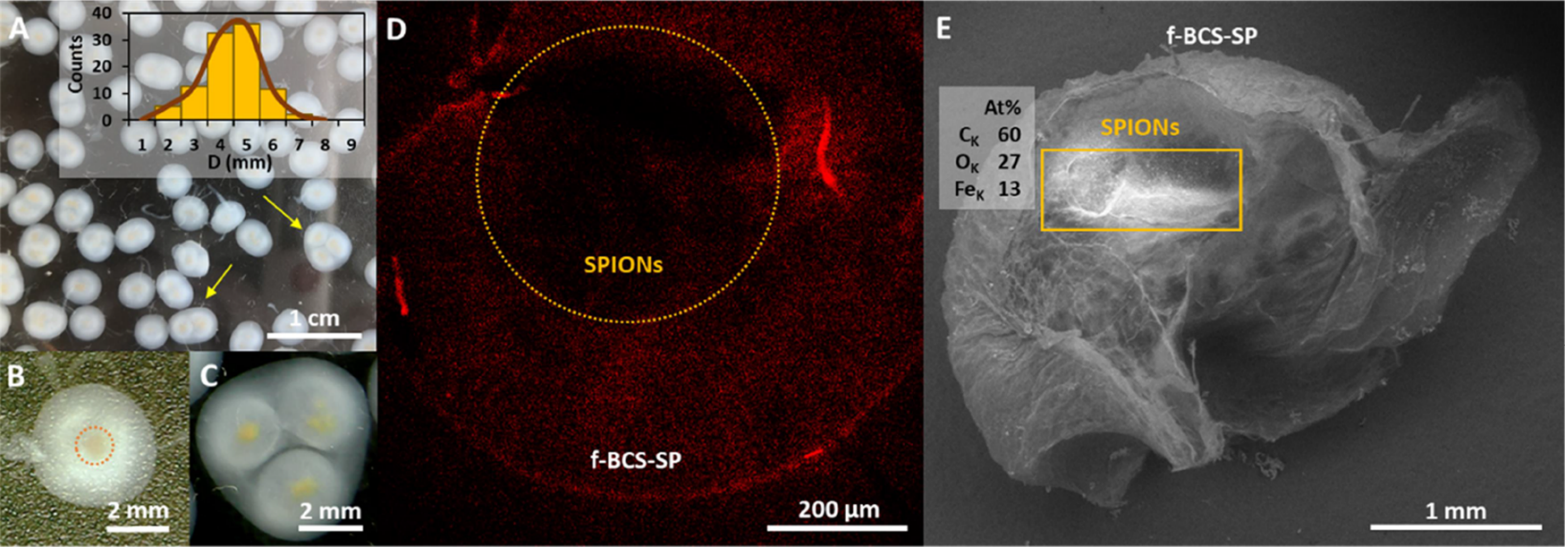
(A) Image of multiple f-BCS-SP produced. Yellow arrows indicate the presence of multispheres. Inset: size histogram with a maximum peak centered at 4 ± 1 mm. (B) Single f-BCS-SP core-shell structure. The magnetic core is delineated by an orange circle. (C) Multisphere f-BCS-SP core-shell structure. (D) Confocal image showing the dark core of the f-BCS-SP because of the presence of SPIONs. (E) SEM picture of a lyophilized f-BCS-SP. The bright area indicates the location of the SPIONs.

In brief, 200 μL of SPIONs (10 mg/mL [SPIONs] in Hestrin-Schramm (HS) medium) was added to 38 mL of culture medium and 2 mL of inoculum. After 3 days at 30 °C in 150 rpm agitation, SPIONs were entrapped in the generated cellulose producing solid spheres of 4 ± 1 mm diameter with a magnetic core (f-BCS-SP) (Figure 2A). Without SPIONs, larger f-BCS with a diameter size of 6 ± 1 mm were obtained (Figure S2). As depicted in Figure 2B, f-BCS-SP presents a brown spot in the center, indicating the presence of SPIONs and the formation of a core-shell structure. Magnetic f-BCS have been previously reported using the agitation methodology with 10 times higher NP concentration;^38^ however, the achieved structure had SPIONs embedded on the whole structure rather than only in the center. This is the first time to our knowledge that core-shell magnetic BCS are produced in one biosynthesis step with such high concentration. A similar core-shell structure was reported for BCS with graphene oxide (GO);^25^ although the concentrations of GO used were lower. The precise size control of the spheres was challenging and secondary structures, that is, spheres embedded within the same structure, were also observed (yellow arrows in Figure 2A,C). This organization confirms that bacteria produce cellulose nanofibers around themselves, embedding the SPIONs during the process. Eventually, all NPs are entrapped and bacteria continue producing cellulose and generating skein-like structures, achieving a core-shell organization. The sustained agitation favors the spheres to physically interact and, in some cases, to coalesce in complex geometries with several spheres embedded. The confocal study depicted in Figure 2D confirmed the filled morphology of the sphere as the fluorescence of the dyed cellulose can be observed in the whole structure, while the black region in the center is attributed to the SPIONs, that are blocking the signal. Scanning electron microscopy (SEM) imaging and energy-dispersive X-ray (EDAX) analysis of a lyophilized f-BCS-SP confirmed the presence of high electron-dense material in the center of the structure identified as SPIONs by the 13% atomic iron presence detected (Figure 2E).

The poor control of the f-BCS-SP structures and the reported decrease in BC yield under agitation^37,39^ motivated the research of alternative strategies. Therefore, we extended the static biosynthesis method developed in our group^40^ to obtain magnetic hollow BC spheres, h-BCS-SP. In brief, 5 μL drops of 1:1 volume ratio SPIONs:bacterial culture were deposited on a hydrophobic surfaces (static water contact angle, WCA = 150 or 86°), and the system was incubated for 3 days at 30 °C in a saturated humid environment. Cellulose grew at the air–liquid drop interface, reproducing the contour of the drop, the shape of which depends on the hydrophobic character of the surface. During growth, SPIONs position and movement were restricted by a magnet, as depicted in Figure 1B.

### Location of SPIONs

**Figure 3:**
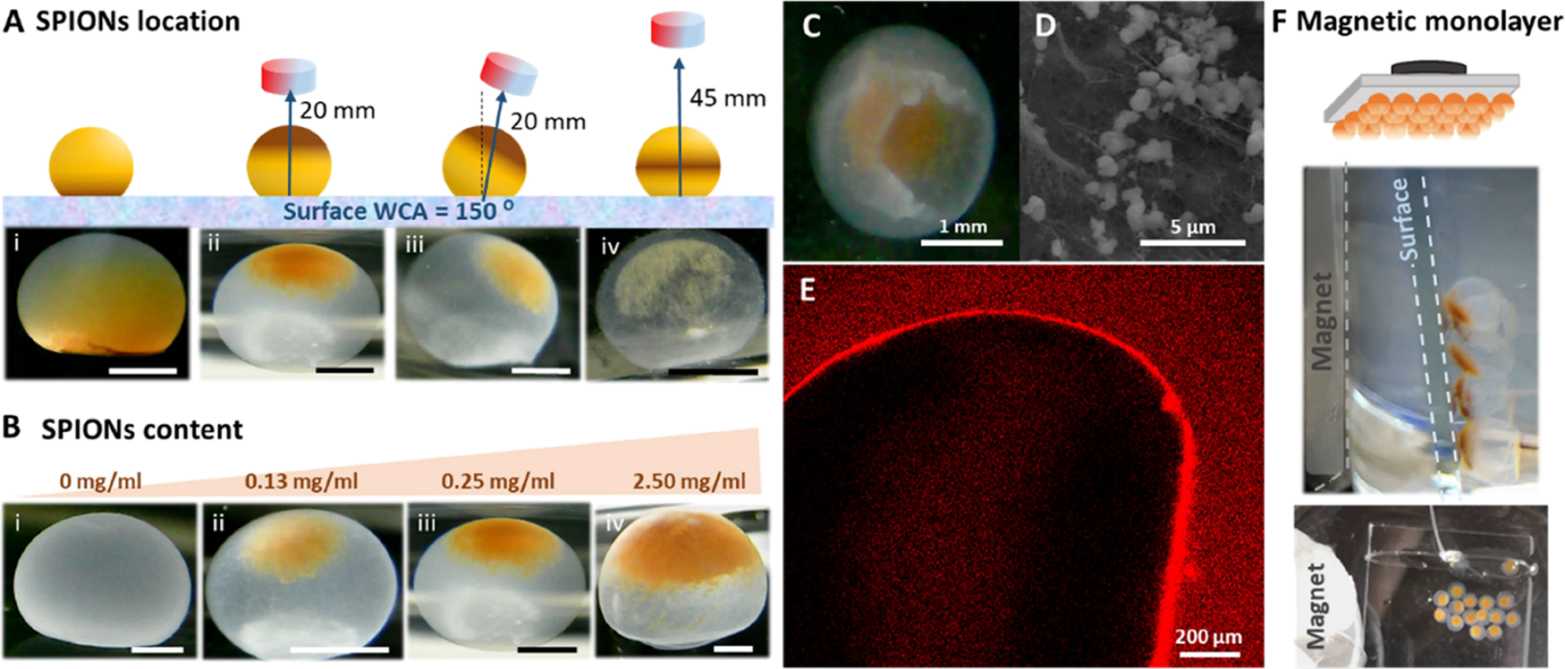
(A) Images of h-BCS-SP under different magnetic field conditions on a superhydrophobic surface (WCA = 150°). Scale bar: 1 mm. (B) h-BCS-SP with different SPION concentrations under the same magnetic field (at 20 mm) on a superhydrophobic surface (WCA = 150°). Scale bar: 1 mm. (C) Broken h-BCS-SP, where the hollow structure and the localized SPIONs loading are clearly seen. (D) SEM picture of the cellulose wall of a lyophilized h-BCS-SP with SPION aggregates on the nanofibers. (E) Confocal image of h-BCS-SP biosynthesized with a magnetic field at 45 mm. In the middle of the sphere, fluorescence is observed because of nanocellulose growth. (F) Example of an h-BCS-SP monolayer ordered arrangement on a glass surface.

SPIONs could be precisely entrapped at different positions of the hollow BCS structure by a local magnetic field produced by a NdFeB permanent magnet. Figure 3A (i–iv) displays the different SPION locations achieved on h-BCS-SP. Easily and reproducibly, we produced magnetic h-BCS structures with SPIONs located on the top spherical cap or in the equatorial area of the structure, just modifying the distance of the magnet from 20 to 45 mm (y-axis). Without a magnetic field, SPIONs precipitated at the bottom of the drop. In all cases, NPs were entangled within the nanocellulose fibers during the biosynthesis, and they remained in the same position after the magnet removal. Figure 3C shows an open h-BCS-SP (20 mm magnet position), exhibiting the hollow structure and confirming the entrapment of SPIONs, which is also corroborated by SEM analysis (Figure 3D). This feature was remarkable in the drops where SPIONs were stably located in the middle of the suggesting that some cellulose is also produced in the center, fastening the NPs. Additionally, if the magnet was tilted in the x-axis at the start of the biosynthesis, it was also possible to displace the final NP position in that direction.

To visualize the internal configuration, spheres were stained by safranin-O and analyzed by confocal microscopy. The production of hollow BC structures was confirmed as the fluorescence signal attributed to the bacterial cellulose was only detected delimiting the sphere, as presented in Figure 3E. Remarkably, for biosynthesized h-BCS-SP with the magnet at 45 mm, we detected cellulose in the equatorial area of the structure, where SPIONs are located. We hypothesize that some bacteria migrate to the SPIONs location attached to them and produce cellulose from that position trying to reach the sphere interface and entrapping the NPs during the process.

### Magnetic Characteristics

As shown in Figure 3B (i–iv), the magnetic loading of the h-BCS-SP was easily controlled by varying the initial SPIONs concentration ([SPIONs]) in the bacterial culture medium, obtaining hollow structures with 0.13, 0.25, and 2.50 mg/mL [SPIONs]. Placing the magnet at 20 mm, we achieved spheres in which the increase in [SPIONs] is directly correlated with the surface covered by NPs, being 26 ± 6% coverage for 0.13 mg/mL [SPIONs], 33 ± 7% for 0.25 mg/mL, and 51 ± 6% for 2.50 mg/mL.

The magnetic character of the composites was analyzed (Figure S3) and a superparamagnetic behavior was observed, indicating that SPIONs do not degrade during the biosynthesis process. In addition, after more than one year in solution, the h-BCS-SP appearance has not changed. h-BCS-SP response in water toward a magnetic field at 10 mm was also directly proportional to the amount of SPIONs. As summarized in Table S1, the 2.50 mg/mL SPION load showed a faster reaction (13.5 mm/s) toward the magnet than 0.25 mg/mL (5.7 mm/s). Figure S4 contains videos displaying the magnetic response. Notably, h-BCS-SP do not change shape after actuation. All these results confirm the strong bond of the NPs into the nanocellulosic matrix with almost negligible leaching of SPIONs; characteristics already reported for BC nanocomposites films.^26,29^ Therefore, as the quantity of SPIONs added in the bacterial culture is all entrapped in the cellulose sphere formed, the amount of magnetic material is fully tunable. Finally, the magnetic manageability of h-BCS-SP was also tested as we assembled them into a single monolayer on a surface, as shown in Figure 3F. This magnetic actuation could facilitate the recovery of the spheres in a solution where they may act as catalysts or switching layers.^41,42^

### Sphericity

**Figure 4:**
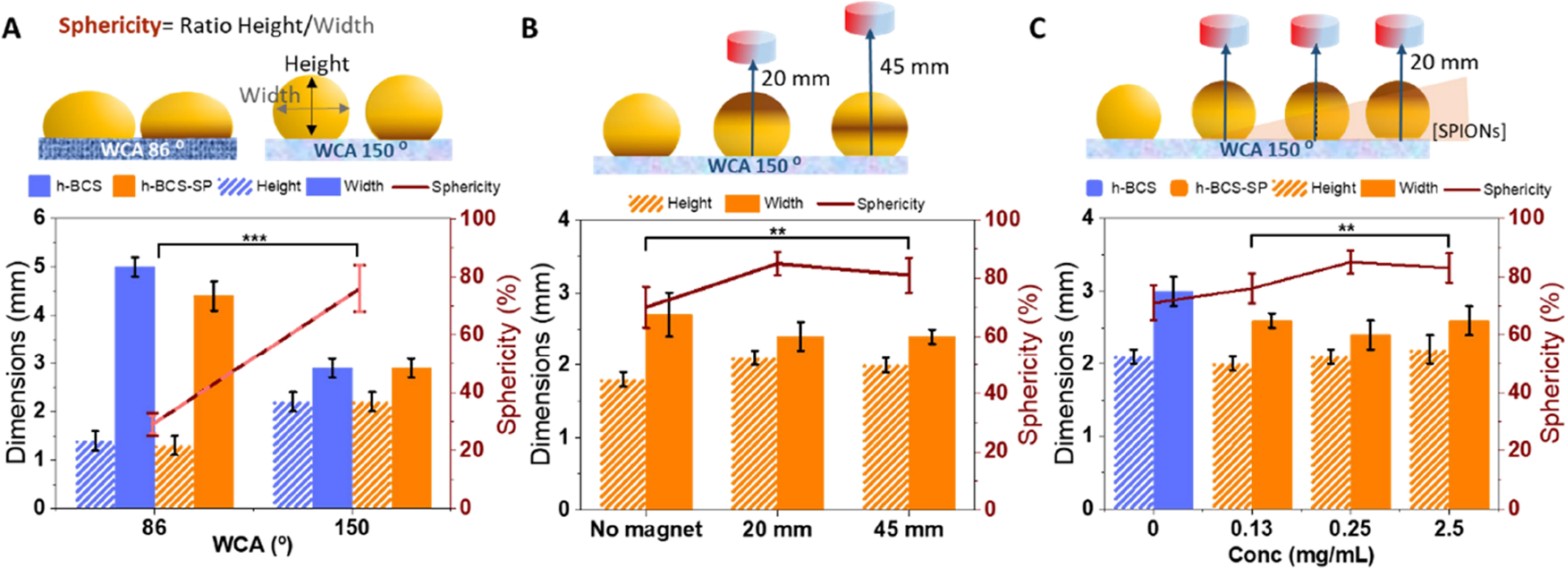
Height and width measurement comparison of the h-BCS structures. (A) Surface hydrophobicity morphological effect on h-BCS with (orange) and without SPIONS (blue). (B) Magnetic field distance effect on the morphology of h-BCS-SP with 0.25 mg/mL [SPIONs]. (C) SPIONs concentration morphological effect on h-BCS-SP production under a magnetic field.

As previously mentioned, the structure shape depends on the hydrophobic character of the culture surface, resulting in hollow spherical structures using superhydrophobic surfaces (WCA = 150°) or hollow dome-like shapes for less hydrophobic surfaces (WCA = 86°).^40^ The percentage of sphericity, defined as the ratio height/width of the structure, being 100% a perfect sphere, was evaluated for magnetic h-BCS. Surfaces with a WCA of 86° produced hollow dome-like shapes with a sphericity of 30%, whereas spheres with approximately 80% sphericity were obtained using surfaces with WCA of 150°, as shown in Figure 4A. Increasing the WCA, the sphericity of the hollow structure increases almost three-fold (*p* < 0.001). The influence of the magnetic field and the [SPIONs] on the morphology of the BCS was also evaluated (Figure 4B,C). Drops with 0.25 mg/mL [SPIONs] grown under a magnetic field (Figure 4B) presented between 10–15% increased sphericity compared to h-BCS-SP cultivated without the magnetic field (*p* < 0.01). However, we detected that the magnet distance does not affect sphericity significantly. The NP concentration was found to also influence the sphericity. When a 0.25 mg/mL of [SPIONs] was used, we computed an increase of 14% sphericity compared to control (i.e., without SPIONs) (Figure 4C). However, at higher [SPIONs], such as 2.50 mg/mL, minimal spherical improvement was observed. Note that in all cases, BC growth was not impeded. Therefore, SPION addition does not affect the bacterial growth and increases the sphericity of the final structures. In light of the results, we hypothesize that as the SPIONs are attracted toward the magnet, the magnetic force applied prevents the drop to flatten, maintaining the spherical shape while, at the same time, the presence of NPs could increase the oxygen availability in the drop.

**Figure 5:**
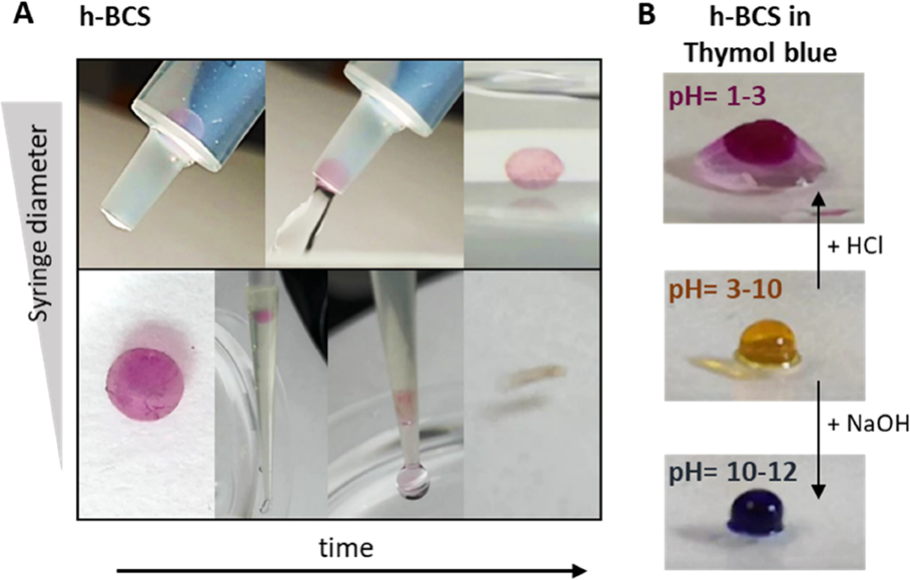
(A) h-BCS images showing the flexibility to pass through a syringe of 2 mm diameter (upper panel) and how it collapses when a 0.4 mm diameter syringe is used (bottom panel). For clarity, h-BCS is dyed with Safranin-O. (B) h-BCS rapid change of color at different pH levels when it contains Thymol Blue.

Flexible behavior is one of the requirements needed for soft actuators to interact with soft tissues and to be non-invasively administered. Hollow and filled BCS flexibility was qualitatively analyzed by squeezing them through syringes with different opening diameters. For clarity, BCS were colored with Thymol Blue and Safranin-O, which allowed us to confirm the preservation of structural integrity. While both structures were able to pass through a 2 mm syringe, only h-BCS recovered its original shape (Figure 5A). We believe that this is mainly due to the flexibility conferred using the hollow internal structure, and therefore, h-BCS show better suitability for soft actuators. However, neither of the BCS were able to pass through a syringe of 0.4 mm and recover its original shape. Figure S5 shows both BCS scenarios for a better comparison.

In addition, we evaluated qualitatively the exchange of fluids within the hollow structures, a characteristic harnessed when a soft actuator is actuated by a chemical reaction. For this, as presented in Figure 5B, the spheres were submerged in thymol blue, which serves as a pH indicator, at pH 6. Once the spheres adsorb the solution and change their color from white-transparent to more yellow, a drop of a concentrated solution of NaOH or HCl was added to produce the color transformation of the indicator. The switch was visible in a few seconds as the color of the BCS changed due to the change in pH.

### Multifunctionality

**Figure 6:**
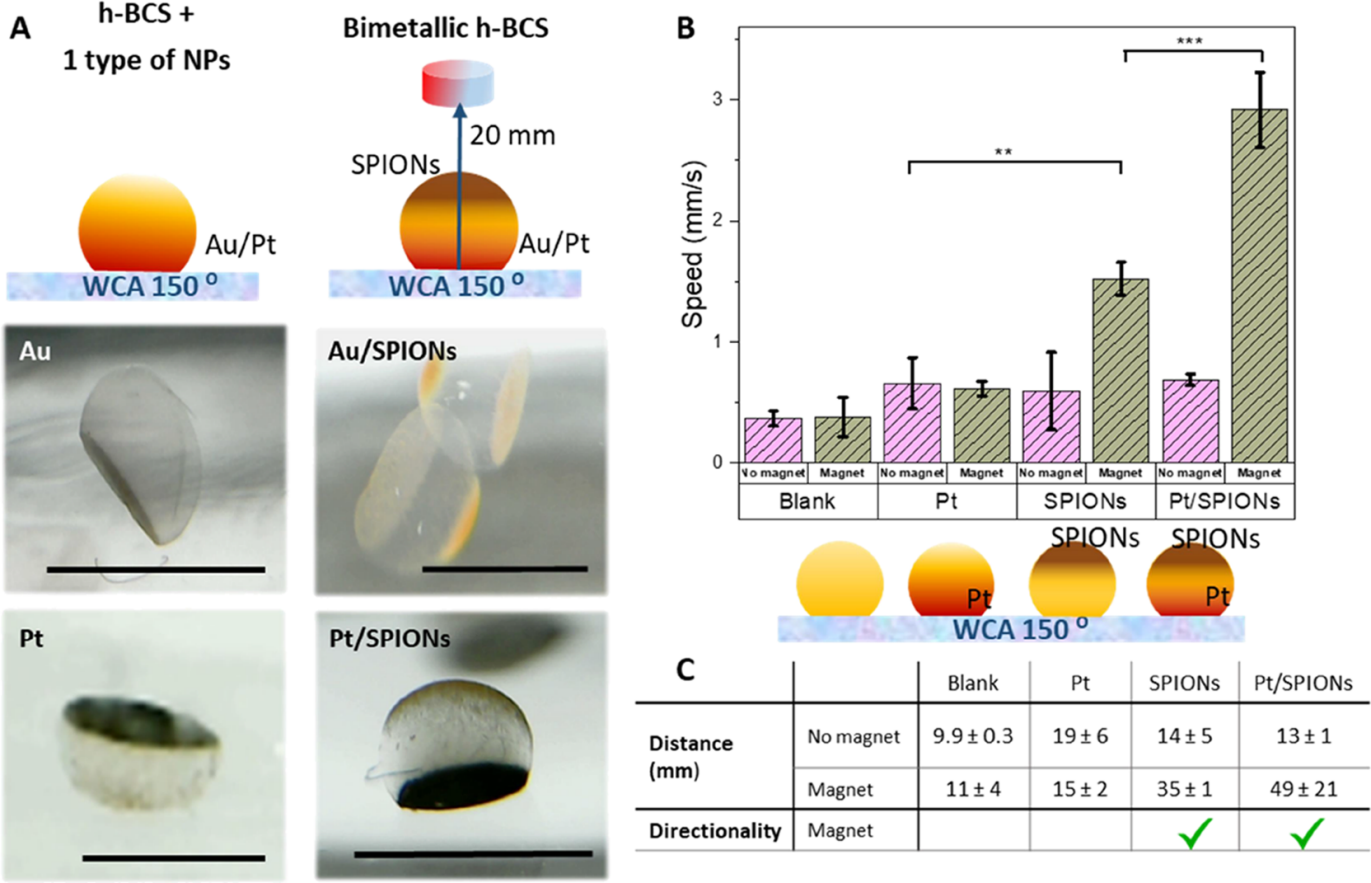
(A) h-BCS structures synthesized with other NPs (left panels) and Janus magnetic h-BCS showing diverse functionalities (right panels). Scale bar: 2 mm. (B) Speed achieved for h-BCS without NPs, with Pt, with SPIONs, or with both in the presence and absence of a magnetic field. (*n* = 3) (C) Table with the distance covered in 30 s and directionality of h-BCS without NPs, with Pt, with SPIONs, or with both in the presence and absence of a magnetic field (*n* = 3). ** *p* < 0.01, *** *p* < 0.001.

The possibility to control the location of the NPs during the biosynthesis on superhydrophobic surfaces allows adding different types of NPs, obtaining BCS with multifunctionalities and even Janus-like structures in a single step by simply using a magnet. In this direction, we created h-BCS-Au and h-BCS-Pt structures adding gold NPs (Au NPs) or platinum NPs (Pt NPs) to the media, as shown in Figure 6A. The bimetallic structures were obtained by controlling the position of SPIONs at the top of the structure using a magnet and embedding Au or Pt NPs at the bottom of the sphere by gravity-driven deposition. This approach reveals new paths to create complex and multifunctional BCS as different types of NPs, concentrations, and parameters of the static culture protocol can be used and controlled to create multifunctional structures on demand.

The hollow Pt/SPIONs Janus structures allow for combining the active movement generated by two forces. On the one hand, external pulled by a magnetic field and, on the other hand, fueled by the catalyzed reaction by the platinum NPs with H_2_O_2_ present in the system. Therefore, spheres were deposited in a water solution with H_2_O_2_ in the presence and absence of a magnetic field, and their aptitude to transform energy into motion, their speed and the ability to control their directionality were evaluated (Figure 6B,C). Directionality was determined as the capability to control the direction of the motion, which was found only for the structures with SPIONs under a magnetic field. The speed was calculated as the distance covered by the sphere in 30 s under the effect of H_2_O_2_ and/or magnetic field. The distance was measured as the path covered by the sphere in 30 s. An enhancement in the distance was observed under a magnetic field for the structures with SPIONs, while the spheres without SPIONs moved in circular trajectories due to Brownian movement and/or catalytic reaction. Directionality and speed were also enhanced almost five-fold for the Janus spheres in comparison to h-BCS-Pt spheres and two-fold when compared to magnetic h-BCS-SP, remarkable results that suggest that the use of the two energy-conversion systems could impact the development of novel soft actuators.

## Conclusions

Two simple, bottom-up biosynthetic methods have been developed and presented to produce magnetic actuable BCS. The agitated bacterial culture process produces filled spheres with a core-shell organization, where the magnetic NPs were located in the center (f-BCS-SP). However, the size and NP loading control of these structures is challenging, obtaining multispheres (spheres embedded on a bigger one). On the contrary, the static method over hydrophobic surfaces allows for the production of hollow structures with high control on the sphere size, location, and concentration of the functional loading within the structure. These spheres have been demonstrated to support NP loadings of approximately half of its volume and to homogeneously integrate and fasten them inside the nanocellulose network.

Both BCS have shown good flexibility, being able to be injected through syringes of 2 mm diameter tip, although only hollow structures recovered the original shape. In addition, both methodologies can be used to produce BCS with functionality on demand. Nonetheless, only h-BCS offers the possibility to produce spheres with located multifunctionality, such as the synthesized Au/SPIONs or Pt/SPIONs hybrids, obtaining Janus bacterial cellulose spheres. The use of a magnetic field and the chemical reaction of H_2_O_2_ on the Janus Pt/SPIONs increased five times the directionality and movement of these structures in comparison to the controls. Thus, the novel biosynthetic procedure described in this work opens up the avenue for unique structures that are otherwise complicated to obtain. We hypothesize that these structures would be applied in fields where silica or collagen spheres are currently used for cargo-delivery systems, theragnostic, tissue engineering, or programmable and self-organizing systems.

## Experimental Section

### Materials

*Komagataeibacter xylinus* (*K. xylinus*) bacterial strain (NCIMB 5346) was provided by the Spanish Type Culture Collection (CECT, Spain). HS culture medium containing 20 g of dextrose, 5 g of peptone, 5 g of yeast extract (Conda Lab.), 6.8 g of sodium phosphate dodecahydrate, and 1.15 g of citric acid monohydrated (Sigma-Aldrich) in 1 L of Milli-Q water was prepared and autoclaved in the laboratory. NaOH (Sigma-Aldrich) 0.1 M solution was employed for cleaning the BCS. SPIONs and gold NPs (AuNPs) were synthesized as previously reported.^29,43^ Platinum NPs (1 mg/mL, 3 nm) and glycerol (≥99.5%) were purchased from Sigma-Aldrich. Superhydrophobic surfaces were produced using our previously published polymer-based method.^44,45^ Neodymium iron boron (NdFeB) magnetic disks (60 × 5 mm) were purchased from Ingeniería Magnética Aplicada, S.L. (IMA), Spain. Safranin-O (Alfa Aesar) was employed to stain the BC fibers for confocal imaging. H_2_O_2_ (30% v/v) from Sigma-Aldrich was used for the directionality analysis.

### Synthesis of f-BCS

*K. xylinus* was inoculated on 6 mL of HS culture medium and incubated statically for 7 days at 30 °C. After shaking and removing the bacterial cellulose pellicle created at the interface, f-BCS were prepared by mixing 2 mL of the bacterial broth with 38 mL of fresh HS medium in a 100 mL conical flask, and the system was placed in an orbital shaker (OVAN, orbital maxi MD) inside the incubator (30 °C, 3 days at 150 rpm). Magnetic f-BCS (f-BCS-SP) were synthesized by adding 200 μL of SPION solution (10 mg/mL [SPIONs] in the HS medium) to the initial culture media volume (38 mL) and stirring to disperse the particles.

Both f-BCS and f-BCS-SP structures were collected by filtration, cleaned by immersion for 10 min into a 50% EtOH in H_2_O solution, and boiled once in Milli-Q water for 40 min and twice in a 0.1 M NaOH solution at 90 °C for 20 min. Finally, they were washed until pH neutralization and kept in Milli-Q water (MQ) until further use.

### Synthesis of h-BCS

h-BCS were prepared by mixing an initial bacterial culture volume with 10% v/v of glycerol to increase the viscosity and avoid deformation due to evaporation during the incubation period. Then, drops of 5 μL were deposited on superhydrophobic slides (WCA of 150°) and Petri dishes (WCA, 86°) as hydrophilic surfaces. h-BCS were incubated for 3 days at 30 °C in a saturated humidity environment and static conditions. Afterward, the spheres were gently removed from the surface with water, cleaned and stored as described before.

To create magnetic actuable h-BCS (h-BCS-SP), before drop deposition, a SPION solution (10 mg/mL in HS medium) was added in a 1:1 ratio to the bacterial culture solution with glycerol. h-BCS-SP with final concentrations of 0.13, 0.25, and 2.50 mg/mL [SPIONs] were obtained in the presence or absence of a magnetic field created using a NdFeB magnetic disk. Applying the same procedure, a 1:1 mixing ratio, Au (2.5 mg/mL HS medium) and Pt (10 mg/mL HS medium) solutions were used to produce h-BCS-Au and h-BCS-Pt, respectively. Bimetallic h-BCS were also achieved using a starting NP solution that contains equal NP proportion in the HS medium.

The static WCA of the surfaces was calculated using a DSA 100 from KRÜSS by depositing a 5 μL drop of culture media on top of the surface and analyzing the contact area with the system software. Three surfaces of each type were tested in three different areas, obtaining a final value of 150° for superhydrophobic surfaces and 86° for Petri dish surfaces.

### Morphological Studies and Statistical Analysis

The morphology of the cellulose spheres was determined by the analysis of digital images with ImageJ software. The size of the f-BCS was computed from populations of 100 to 200 spheres. h-BCS height and width average values were obtained for sample populations of 6 to 21 spheres, and the spherical ratio (sphericity) was computed as the height/width ratio. One-way analysis of variance (ANOVA), followed by Tukey’s multiple comparison test, was used for statistical analysis. Statistical significance was accepted at 0.05.

### Transmission Electron Microscopy

The different NP systems used in this work were analyzed with a 120 kV JEOL JEM-1210 transmission electron microscope. NPs mean size was calculated from measurements of population of 300 to 400 particleswith ImageJ software. Polydispersity index (PDI) was calculated as the percentage of the standard deviation/mean value. The selected-area electron diffraction (SAED) mode was used to corroborate the structure of the NPs (Figure S1).

### Scanning Electron Microscopy

Prior to SEM analysis, spheres were lyophilized to maintain the structure upon drying. The spheres were submerged with the smallest amount of MQ water needed inside a vial. Then, the vial was sealed with pierced aluminum paper and placed inside a lyophilizer (LYOQUEST-85) at −80 °C and 0.05 mBar for 48 h. After drying, samples were placed on a SEM aluminum holder over a carbon tape adhesive. Images were taken with a QUANTA FEI 200 FEG-ESEM under low-vacuum conditions, an acceleration voltage of 10–15 kV, an electron beam spot of 3.5, and a working distance of 10.0 mm. Elemental scanning performed with an electron-dispersive X-ray equipment corroborated the presence or absence of NPs inside the cellulose macrostructures.

### Confocal Microscopy

The sphere internal structure was evaluated by confocal microscopy. BCS were stained 12 h with 1 mL of Safranin-O solution (0.5 mM) and rinsed several times with MQ water. Then, a single sphere was placed in a hand-made holder that consists of a glass cover where a plastic washer was glued, and the extra water was removed. A laser of HeNe (λ = 570 nm) was used to excite Safranin-O. Multistack confocal images were obtained with a Leica TCS SP5 at the Centre for Research in Agricultural Genomics (CRAG, Spain) under the following conditions: 1.55 × 1.55 format, pinhole 1, zoom 1 bidirectional, 10× magnification, a step size of 0.8 μm, a maximum size of 1.5 mm, and a speed of 600 Hz. ImageJ software was used for image processing.

### Directionality and Speed Analysis of Janus Structures

The speed response toward a magnetic field correlated with the SPION concentration on the h-BCS structures was computed for magnetic h-BCS (h-BCS-SP). A sphere was placed in the middle of a vessel with water and with a magnet placed at the vessel external wall. The distance covered by the sphere (10 mm) was computed considering *t* = 0 the moment at which the drop orientates towards the magnet, and the final time when it reached the glass wall in contact with the magnet. The measurement was replicated three times for each SPION concentration.

The directionality toward a magnetic field of h-BCS, h-BCS-Pt, h-BCS-SP, and h-BCS Pt/SPIONs was measured by placing each BCS typein a 4:1 H_2_O:H_2_O_2_ ratio solution at 24 mm from a magnet, and their movement was recorded for 30 s. Tracking of the spheres toward the magnet was performed manually with the tracking plugin from the ImageJ software. Sample measurements were performed three times. One-way ANOVA followed by Tukey’s multiple comparison test was used for statistical analysis. Statistical significance , *p* > 0.05.

### Superconducting Quantum Interface Device

The magnetization of h-BCS-SP was analyzed with a Quantum Design MPMS-XL equipment. A sphere with 2.50 mg/mL SPIONs concentration was dried in the middle of a paper strip at room temperature. The strip was then placed inside a plastic tube with a similar diameter and sealed with cotton. Magnetization vs applied magnetic field was measured from 0 to 70 kOe at 300 K, and the magnetic response of the SPIONs trapped in the cellulose sphere was obtained.

## Supporting Information Available

Additional data including NP characterization (TEM, SAED, and histogram); full-BCS size histogram comparison with and without SPIONs; magnetic response data and videos of h-BCS-SP at different concentrations; SQUID data; and squeezing test of the full and hollow spheres (PDF)
Video displaying the magnetic response of a a drop BC-SPIONS with a SPIONS load of 0.13 mg/mL (MP4)
Video displaying the magnetic response of a a drop BC-SPIONS with a SPIONS load of 0.25 mg/mL (MP4)
Video displaying the magnetic response of a a drop BC-SPIONS with a SPIONS load of 2.5 mg/mL (MP4)

## References

[ref1] Zeng M.; Laromaine A.; Roig A. Bacterial Cellulose Films: Influence of Bacterial Strain and Drying Route on Film Properties. Cellulose 2014, 21, 4455–4469. 10.1007/s10570-014-0408-y.

[ref2] Wang S.; Jiang F.; Xu X.; Kuang Y.; Fu K.; Hitz E.; Hu L. Super-Strong, Super-Stiff Macrofibers with Aligned, Long Bacterial Cellulose Nanofibers. Adv. Mater. 2017, 29, 1702498 10.1002/adma.201702498. 28731208

[ref3] Anton-Sales I.; D’Antin J. C.; Fernández-Engroba J.; Charoenrook V.; Laromaine A.; Roig A.; Michael R. Bacterial Nanocellulose as a Corneal Bandage Material: A Comparison with Amniotic Membrane. Biomater. Sci. 2020, 8, 2921–2930. 10.1039/d0bm00083c. 32314754

[ref4] Agrahari V.; Agrahari V.; Chou M.-L.; Chew C. H.; Noll J.; Burnouf T. Intelligent Micro-/Nanorobots as Drug and Cell Carrier Devices for Biomedical Therapeutic Advancement: Promising Development Opportunities and Translational Challenges. Biomaterials 2020, 260, 120163 10.1016/j.biomaterials.2020.120163. 32882512

[ref5] Fusco S.; Huang H.-W.; Peyer K. E.; Peters C.; Häberli M.; Ulbers A.; Spyrogianni A.; Pellicer E.; Sort J.; Pratsinis S. E.; Nelson B. J.; Sakar M. S.; Pané S. Shape-Switching Microrobots for Medical Applications: The Influence of Shape in Drug Delivery and Locomotion. ACS Appl. Mater. Interfaces 2015, 7, 6803–6811. 10.1021/acsami.5b00181. 25751020

[ref6] Hu W.; Lum G. Z.; Mastrangeli M.; Sitti M. Small-Scale Soft-Bodied Robot with Multimodal Locomotion. Nature 2018, 554, 81–85. 10.1038/nature25443. 29364873

[ref7] Vach P. J.; Brun N.; Bennet M.; Bertinetti L.; Widdrat M.; Baumgartner J.; Klumpp S.; Fratzl P.; Faivre D. Selecting for Function: Solution Synthesis of Magnetic Nanopropellers. Nano Lett. 2013, 13, 5373–5378. 10.1021/nl402897x. 24127909 PMC3885197

[ref8] Wang H.; Pumera M. Emerging Materials for the Fabrication of Micro/Nanomotors. Nanoscale 2017, 9, 2109–2116. 10.1039/c6nr09217a. 28144663

[ref9] Liang Y.; Wang H.; Yao D.; Chen Y.; Deng Y.; Wang C. Transportation and Release of Janus Micromotors by Two-Stage Rocket Hydrogel. J. Mater. Chem. A 2017, 5, 18442–18447. 10.1039/c7ta06032g.

[ref10] Wang S.; Liu X.; Wang Y.; Xu D.; Liang C.; Guo J.; Ma X. Biocompatibility of Artificial Micro/Nanomotors for Use in Biomedicine. Nanoscale 2019, 11, 14099–14112. 10.1039/c9nr03393a. 31214671

[ref11] Ceylan H.; Yasa I. C.; Yasa O.; Tabak A. F.; Giltinan J.; Sitti M. 3D-Printed Biodegradable Microswimmer for Theranostic Cargo Delivery and Release. ACS Nano 2019, 13, 3353–3362. 10.1021/acsnano.8b09233. 30742410 PMC6728090

[ref12] Srivastava S. K.; Ajalloueian F.; Boisen A. Thread-like Radical-Polymerization via Autonomously Propelled (TRAP) Bots. Adv. Mater. 2019, 31, 1901573 10.1002/adma.201901573. 31165526

[ref13] Huang H.-W.; Uslu F. E.; Katsamba P.; Lauga E.; Sakar M. S.; Nelson B. J. Adaptive Locomotion of Artificial Microswimmers. Sci. Adv. 2019, 5, eaau1532 10.1126/sciadv.aau1532. 30746446 PMC6357760

[ref14] Dong M.; Wang X.; Chen X.-Z.; Mushtaq F.; Deng S.; Zhu C.; Torlakcik H.; Terzopoulou A.; Qin X.-H.; Xiao X.; Puigmartí-Luis J.; Choi H.; Pêgo A. P.; Shen Q.-D.; Nelson B. J.; Pané S. 3D-Printed Soft Magnetoelectric Microswimmers for Delivery and Differentiation of Neuron-like Cells. Adv. Funct. Mater. 2020, 30, 1910323 10.1002/adfm.201910323.

[ref15] Apsite I.; Biswas A.; Li Y.; Ionov L. Microfabrication Using Shape-Transforming Soft Materials. Adv. Funct. Mater. 2020, 30, 1908028 10.1002/adfm.201908028.

[ref16] Croissant J.; Zink J. I. Nanovalve-Controlled Cargo Release Activated by Plasmonic Heating. J. Am. Chem. Soc. 2012, 134, 7628–7631. 10.1021/ja301880x. 22540671 PMC3800183

[ref17] Su X.; Gupta I.; Jonnalagadda U. S.; Kwan J. J. Complementary Effects of Porosigen and Stabilizer on the Structure of Hollow Porous Poly(Lactic-Co-Glycolic Acid) Microparticles. ACS Appl. Polym. Mater. 2020, 2, 3696–3703. 10.1021/acsapm.0c00696.

[ref18] Zhang Q.; Qin M.; Zhou X.; Nie W.; Wang W.; Li L.; He C. Porous Nanofibrous Scaffold Incorporated with S1P Loaded Mesoporous Silica Nanoparticles and BMP-2 Encapsulated PLGA Microspheres for Enhancing Angiogenesis and Osteogenesis. J. Mater. Chem. B 2018, 6, 6731–6743. 10.1039/c8tb02138d. 32254690

[ref19] Chatterjee S.; Li X. S.; Liang F.; Yang Y. W. Design of Multifunctional Fluorescent Hybrid Materials Based on SiO2 Materials and Core–Shell Fe3O4@SiO2 Nanoparticles for Metal Ion Sensing. Small 2019, 15, 1904569 10.1002/smll.201904569. 31573771

[ref20] Tao J.; Su X.; Li J.; Shi W.; Teng Z.; Wang L. Intricately Structured Mesoporous Organosilica Nanoparticles: Synthesis Strategies and Biomedical Applications. Biomater. Sci. 2021, 9, 1609–1626. 10.1039/d0bm02157a. 33459311

[ref21] Meng C.; Hu J.; Gourlay K.; Yu C.; Saddler J. N. Controllable Synthesis Uniform Spherical Bacterial Cellulose and Their Potential Applications. Cellulose 2019, 26, 8325–8336. 10.1007/s10570-019-02446-5.

[ref22] Mashkour M.; Rahimnejad M.; Mashkour M.; Soavi F. Electro-Polymerized Polyaniline Modified Conductive Bacterial Cellulose Anode for Supercapacitive Microbial Fuel Cells and Studying the Role of Anodic Biofilm in the Capacitive Behavior. J. Power Sources 2020, 478, 228822 10.1016/j.jpowsour.2020.228822.

[ref23] Pham T. T. H.; Vadanan S. V.; Lim S. Enhanced Rheological Properties and Conductivity of Bacterial Cellulose Hydrogels and Aerogels through Complexation with Metal Ions and PEDOT/PSS. Cellulose 2020, 27, 8075–8086. 10.1007/s10570-020-03284-6.

[ref24] Yuan Q.; Li L.; Peng Y.; Zhuang A.; Wei W.; Zhang D.; Pang Y.; Bi X. Biomimetic Nanofibrous Hybrid Hydrogel Membranes with Sustained Growth Factor Release for Guided Bone Regeneration. Biomater. Sci. 2021, 9, 1256 10.1039/d0bm01821j. 33470265

[ref25] Urbina L.; Eceiza A.; Gabilondo N.; Corcuera M. Á.; Retegi A. Tailoring the in Situ Conformation of Bacterial Cellulose-Graphene Oxide Spherical Nanocarriers. Int. J. Biol. Macromol. 2020, 163, 1249–1260. 10.1016/j.ijbiomac.2020.07.077. 32673723

[ref26] Abol-Fotouh D.; Dörling B.; Zapata-Arteaga O.; Rodríguez-Martínez X.; Gómez A.; Reparaz J. S.; Laromaine A.; Roig A.; Campoy-Quiles M. Farming Thermoelectric Paper. Energy Environ. Sci. 2019, 12, 716–726. 10.1039/c8ee03112f. 30930961 PMC6394882

[ref27] Wan Y. Z.; Huang Y.; Yuan C. D.; Raman S.; Zhu Y.; Jiang H. J.; He F.; Gao C. Biomimetic Synthesis of Hydroxyapatite/Bacterial Cellulose Nanocomposites for Biomedical Applications. Mater. Sci. Eng. C 2007, 27, 855–864. 10.1016/j.msec.2006.10.002.

[ref28] Li G.; Zou C.; Sun Y.; Fan W.; Ma X.; Tao J.; Li P.; Xu Y. Nonfreeze-Drying Approach for Anisotropic Compression-Resilient Inorganic Aerogels by Guided Self-Assembly and Controlled Mineralization of Bacterial Cellulose. ACS Sustainable Chem. Eng. 2019, 7, 14591–14600. 10.1021/acssuschemeng.9b02195.

[ref29] Roig-Sanchez S.; Jungstedt E.; Anton-Sales I.; Malaspina D. C.; Faraudo J.; Berglund L. A.; Laromaine A.; Roig A. Nanocellulose Films with Multiple Functional Nanoparticles in Confined Spatial Distribution. Nanoscale Horiz. 2019, 4, 634–641. 10.1039/c8nh00310f.

[ref30] Sriplai N.; Pinitsoontorn S. Bacterial Cellulose-Based Magnetic Nanocomposites: A Review. Carbohydr. Polym. 2021, 254, 117228 10.1016/j.carbpol.2020.117228. 33357842

[ref31] Shah N.; Ul-Islam M.; Khattak W. A.; Park J. K. Overview of Bacterial Cellulose Composites: A Multipurpose Advanced Material. Carbohydr. Polym. 2013, 98, 1585–1598. 10.1016/j.carbpol.2013.08.018. 24053844

[ref32] Schaffner M.; Rühs P. A.; Coulter F.; Kilcher S.; Studart A. R. 3D Printing of Bacteria into Functional Complex Materials. Sci. Adv. 2017, 3, eaao6804 10.1126/sciadv.aao6804. 29214219 PMC5711516

[ref33] Yu J.; Huang T. R.; Lim Z. H.; Luo R.; Pasula R. R.; Liao L. D.; Lim S.; Chen C. H. Production of Hollow Bacterial Cellulose Microspheres Using Microfluidics to Form an Injectable Porous Scaffold for Wound Healing. Adv. Healthcare Mater. 2016, 5, 2983–2992. 10.1002/adhm.201600898. 27805793

[ref34] Amin M. C. I. M.; Abadi A. G.; Katas H. Purification, Characterization and Comparative Studies of Spray-Dried Bacterial Cellulose Microparticles. Carbohydr. Polym. 2014, 99, 180–189. 10.1016/j.carbpol.2013.08.041. 24274495

[ref35] Hoshi T.; Endo M.; Hirai A.; Suzuki M.; Aoyagi T. Encapsulation of Activated Carbon into a Hollow-Type Spherical Bacterial Cellulose Gel and Its Indole-Adsorption Ability Aimed at Kidney Failure Treatment. Pharmaceutics 2020, 12, 1076 10.3390/pharmaceutics12111076. PMC7696591 33187079

[ref36] Hu Y.; Catchmark J. M.; Vogler E. A. Factors Impacting the Formation of Sphere-like Bacterial Cellulose Particles and Their Biocompatibility for Human Osteoblast Growth. Biomacromolecules 2013, 14, 3444–3452. 10.1021/bm400744a. 24010638

[ref37] Hu Y.; Catchmark J. M. Formation and Characterization of Spherelike Bacterial Cellulose Particles Produced by Acetobacter Xylinum JCM 9730 Strain. Biomacromolecules 2010, 11, 1727–1734. 10.1021/bm100060v. 20518455

[ref38] Zhu H.; Jia S.; Wan T.; Jia Y.; Yang H.; Li J.; Yan L.; Zhong C. Biosynthesis of Spherical Fe3O4/Bacterial Cellulose Nanocomposites as Adsorbents for Heavy Metal Ions. Carbohydr. Polym. 2011, 86, 1558–1564. 10.1016/j.carbpol.2011.06.061.

[ref39] Brandes R.; de Souza L.; Vanin D. V. F.; Carminatti C. A.; Oliveira E. M.; Antônio R. V.; Recouvreux D. O. S. Influence of the Processing Parameters on the Characteristics of Spherical Bacterial Cellulose. Fibers Polym. 2018, 19, 297–306. 10.1007/s12221-018-7679-5.

[ref40] Laromaine A.; Tronser T.; Pini I.; Parets S.; Levkin P. A.; Roig A. Free-Standing Three-Dimensional Hollow Bacterial Cellulose Structures with Controlled Geometry via Patterned Superhydrophobic-Hydrophilic Surfaces. Soft Matter 2018, 14, 3955–3962. 10.1039/c8sm00112j. 29736513

[ref41] Chen M.; Kang H.; Gong Y.; Guo J.; Zhang H.; Liu R. Bacterial Cellulose Supported Gold Nanoparticles with Excellent Catalytic Properties. ACS Appl. Mater. Interfaces 2015, 7, 21717–21726. 10.1021/acsami.5b07150. 26357993

[ref42] Wang Q.; Tian D.; Hu J.; Huang M.; Shen F.; Zeng Y.; Yang G.; Zhang Y.; He J. Harvesting Bacterial Cellulose from Kitchen Waste to Prepare Superhydrophobic Aerogel for Recovering Waste Cooking Oil toward a Closed-Loop Biorefinery. ACS Sustainable Chem. Eng. 2020, 8, 13400–13407. 10.1021/acssuschemeng.0c04212.

[ref43] Zhao L.; Jiang D.; Cai Y.; Ji X.; Xie R.; Yang W. Tuning the Size of Gold Nanoparticles in the Citrate Reduction by Chloride Ions. Nanoscale 2012, 4, 5071–5076. 10.1039/c2nr30957b. 22776896

[ref44] Feng W.; Li L.; Ueda E.; Li J.; Heißler S.; Welle A.; Trapp O.; Levkin P. A. Surface Patterning via Thiol-Yne Click Chemistry: An Extremely Fast and Versatile Approach to Superhydrophilic-Superhydrophobic Micropatterns. Adv. Mater. Interfaces 2014, 1, 1400269 10.1002/admi.201400269.

[ref45] Popova A. A.; Demir K.; Hartanto T. G.; Schmitt E.; Levkin P. A. Droplet-Microarray on Superhydrophobic-Superhydrophilic Patterns for High-Throughput Live Cell Screenings. RSC Adv. 2016, 6, 38263–38276. 10.1039/c6ra06011k.

