# Supplementary material for: One-Step Biosynthesis of Soft Magnetic Bacterial Cellulose Spheres with Localized Nanoparticle Functionalization
Source: ACS Appl Mater Interfaces. 2021 Nov 12;13(46):55569–76. doi: 10.1021/acsami.1c17752 (PMC8631704; doi:10.1021/acsami.1c17752)

Supporting information

One-step biosynthesis of soft magnetic bacterial cellulose spheres with localized nanoparticles functionalization

Soledad Roig-Sanchez*[1], Oriol Torrecilla*[1], Jordi Floriach-Clark[1], Sebastià Parets[1], Pavel A. Levkin[2], Anna Roig+[1], Anna Laromaine+[1]

[1] Institut de Ciència de Materials de Barcelona, ICMAB-CSIC, Campus UAB, 08193 Bellaterra, Spain

[2] Institute of Biological and Chemical Systems-Functional Molecular Systems (IBCS-FMS), Karlsruhe Institute of Technology (KIT), 76344, Eggenstein-Leopoldshafen, Germany

* Authors contributed equally to the work

+ Corresponding authors: alaromaine@icmab.es

Figure S1: Nanoparticle characterization. A) TEM picture of the nanoparticles synthesized in-house (Fe_2O_3 and Au) and commercially available (Pt). B) SAED images of the different systems where the characteristic crystallographic planes are highlighted. C) Particle size distribution computed from TEM images.

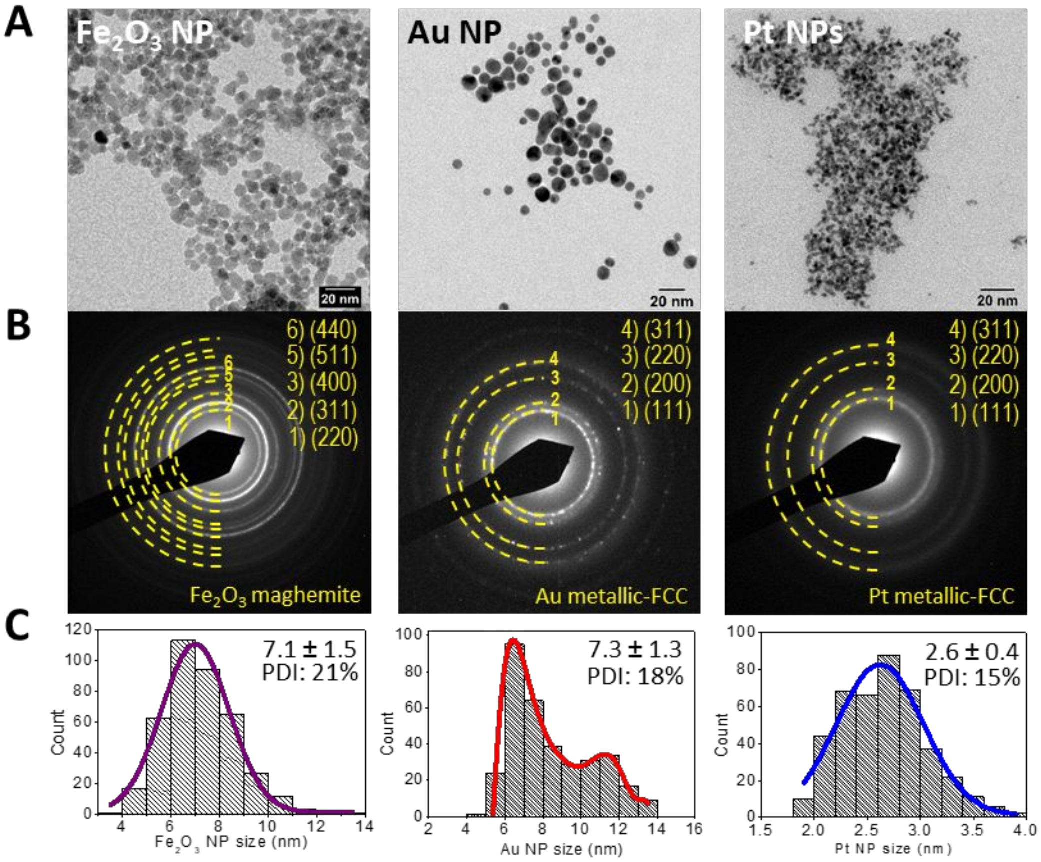

Figure S2: A) f-BCS-SP size histogram showing a maximum peak at 4 ± 1 mm (n = 95). B) f-BCS size histogram showing a maximum peak at 6 ± 1 mm (n= 185).

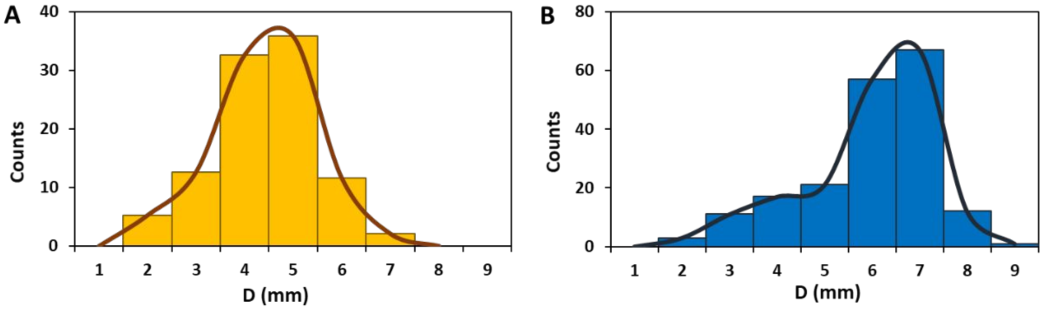

Table S1: Speed response of h-BCS-SP with different SPIONs concentrations towards a magnetic field applied.

Magnetic response	
**[SPIONs] (mg/mL)**	**Speed (mm/s)**
0.13	5.7 ± 1.4
0.25	7.5 ± 0.3
2.50	13.5 ± 1.7

Figure S3: Magnetization vs. applied magnetic field at 300K of h-BCS-SP with 2.50 mg/mL SPIONs.

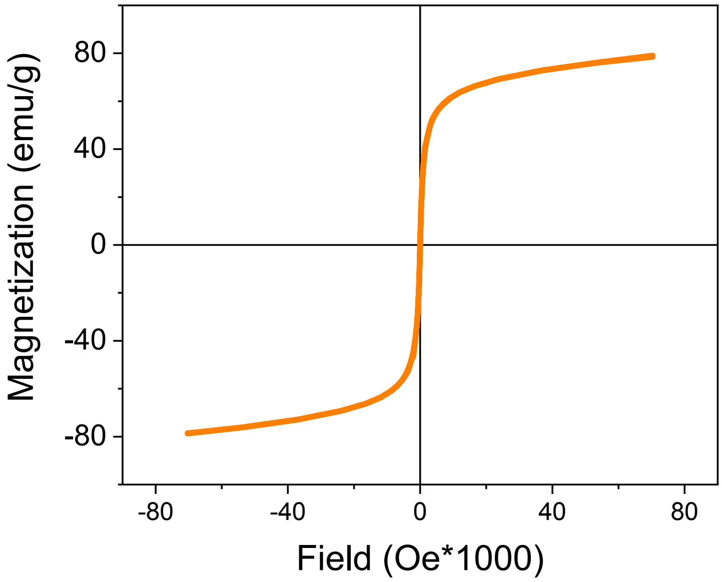

Figure S4: Videos showing the different speed orientation and movement of h-BCS-SP with different SPIONs concentration.

Figure S5: Images showing the shape change of A) f-BCS and B) h-BCS, after being squeezed through a 2 mm syringe diameter (upper panel) and a 0.4 mm syringe diameter (bottom panel). For a better clarity BCS were dyed with Thymol blue and Safranin-O. Scale bar: 1 mm.

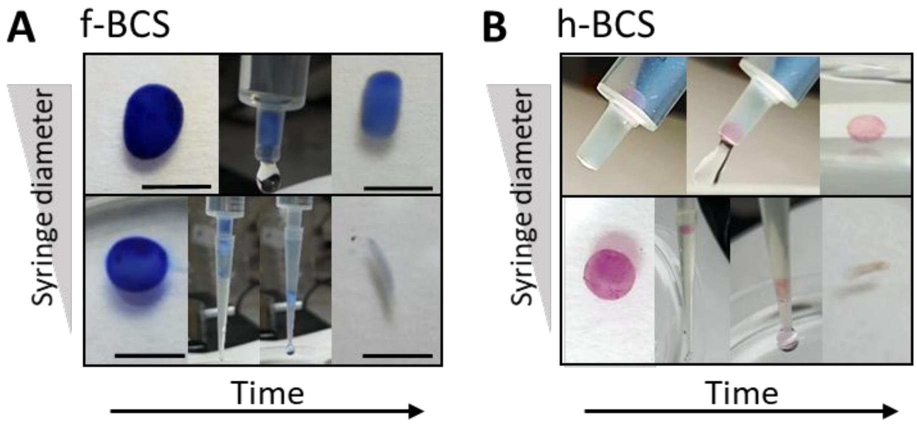

Supplement: Supplementary file 1 — am1c17752_si_001.pdf [file am1c17752_si_001.pdf]
